# Non-Invasive Sensors Integration for NCDs with AIoT Based Telemedicine System

**DOI:** 10.3390/s24144431

**Published:** 2024-07-09

**Authors:** Chavis Srichan, Pobporn Danvirutai, Noppakun Boonsim, Ariya Namvong, Chayada Surawanitkun, Chanachai Ritsongmuang, Apirat Siritaratiwat, Sirirat Anutrakulchai

**Affiliations:** 1 Faculty of Engineering, Khon Kaen University, Khon Kaen 40002, Thailand; apirat@kku.ac.th; 2 Chronic Kidney Disease Prevention in Northeast Thailand, Khon Kaen University, Khon Kaen 40002, Thailand; pobporn.d@kkumail.com (P.D.); sirirt_a@kku.ac.th (S.A.); 3 Faculty of Interdisciplinary Studies, Khon Kaen University, Nong Khai Campus, Nong Khai 43000, Thailand; boonsim@kku.ac.th (N.B.); ariyna@kku.ac.th (A.N.); chaysu@kku.ac.th (C.S.); 4 Center of Multidisciplinary Innovation Network Talent (MINT Center), Department of Technology and Engineering, Faculty of Interdisciplinary Studies, Khon Kaen University, Nong Khai Campus, Nong Khai 43000, Thailand; 5 Bureau of Academic Services, Khon Kaen University, Khon Kaen 40002, Thailand; chanri@kku.ac.th; 6 Faculty of Medicine, Khon Kaen University, Khon Kaen 40002, Thailand

**Keywords:** non-invasive sensors, telemedicine, data mining, point of care, telehealth, eHealth, biomedical engineering, long short-term memory, telemedicine kiosks, noncommunicable diseases

## Abstract

Thailand’s hospitals face overcrowding, particularly with non-communicable disease (NCD) patients, due to a doctor shortage and an aging population. Most literature showed implementation merely on web or mobile application to teleconsult with physicians. Instead, in this work, we developed and implemented a telemedicine health kiosk system embedded with non-invasive biosensors and time-series predictors to improve NCD indicators over an eight-month period. Two cohorts were randomly selected: a control group with usual care and a telemedicine-using group. The telemedicine-using group showed significant improvements in average fasting blood glucose (148 to 130 mg/dL) and systolic blood pressure (152 to 138 mmHg). Data mining with the Apriori algorithm revealed correlations between diseases, occupations, and environmental factors, informing public health policies. Communication between kiosks and servers used LoRa, 5G, and IEEE802.11, which are selected based on the distance and signal availability. The results support telemedicine kiosks as effective for NCD management, significantly improving key NCD indicators, average blood glucose, and blood pressure.

## 1. Introduction

Rural healthcare in Thailand faces significant challenges, including poverty, high transportation costs, and lengthy travel times to distant hospitals. These obstacles are compounded by a low doctor-to-population ratio, particularly in rural areas, leading to hospital congestion and increased risks of infectious disease exposure. Addressing these issues is crucial to improving healthcare accessibility and outcomes for rural populations. The doctor-to-population ratio in Thailand is 1:1680, and it is likely worse in rural areas, leading to hospital congestion and increased risk of infectious disease exposure. For example, SARS-CoV-2 infection in NCD patients can result in higher mortality rates [1]. To enhance healthcare accessibility and reduce costs, “Suksala Temed”, a telemedicine health kiosk aimed at improving NCD healthcare in remote areas, was implemented in Northeast Thailand. This initiative helps avoid hospital congestion and reduces the risk of airborne disease exposure, like SARS-CoV-2 [2].

Several telemedicine frameworks have been proposed and studied [3,4,5,6,7,8,9]; however, few implementations have been reported [10,11]. We collected statistics from the population in the five sub-districts where kiosks were distributed (N = 2954; 1210 controlled and 1844 telemedicine-using volunteers). Our study showed that Suksala Temed resulted in significant improvements in patients with NCDs, as evidenced by the improved mean values of fasting blood glucose and systolic blood pressure in diabetes and hypertension patients.

Telemedicine is gaining popularity in Thailand as the country aims to provide healthcare access, particularly in rural areas with a very low doctor-to-patient ratio [12]. Our work focuses on incorporating non-invasive biomedical sensors into telemedicine kiosks to enable rapid screening and preventive care. These sensors monitor blood glucose levels, kidney injury biomarkers, and blood pressure, providing real-time data for remote analysis and monitoring. Additionally, we implemented time-series prediction for blood glucose and blood pressure levels using a long short-term memory (LSTM) model, enabling proactive health management.

In our previous research on biomedical sensors, we developed non-invasive sensors for real-time monitoring of glucose levels [13] and for detecting acute kidney injury from urine biomarkers [14,15]. These sensors provide continuous measurement for each patient, enabling the detection of individual symptom improvements or deterioration trends. Although non-invasive sensors may have lower precision and accuracy than invasive approaches, their clinical acceptability has been demonstrated in various cases, such as in glucose sensors [13].

**Figure 1:**
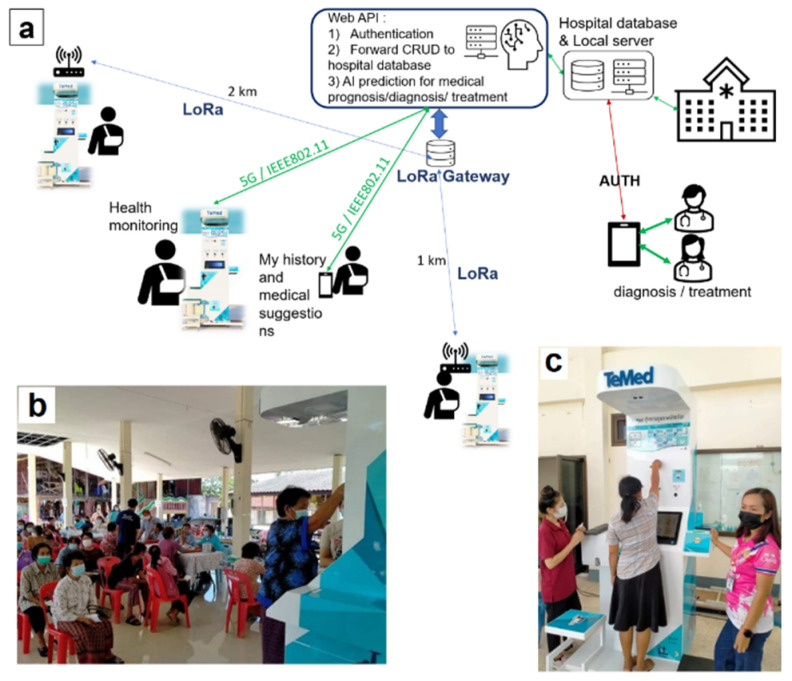
(**a**) Big picture of designed and implemented “Suksala Temed” telemedicine kiosk systems; (**b**,**c**) people using the “Suksala Temed” kiosk.

The telemedicine system (Figure 1) comprises the local hospital server managing outpatient records and connecting with the cloud API (Application Programming Interface), which handles requests from telemedicine kiosks. Communication protocols include Long Range communications (LoRa) for distant rural areas without internet access, and 5G or IEEE802.11 for nearer distances. Doctors must go through authorization to access patient records, including additional records collected from non-invasive sensors and their associated AI prognosis predictions. Using these data, physicians can access broader and more frequent health information, as patients can visit the kiosks and take measures of blood pressure and blood glucose more often than going to the hospital. Therefore, doctors can make more suitable decisions regarding effective treatment.

The telemedicine system presents a crucial solution for improving healthcare accessibility in rural Thailand. Our study demonstrated that telemedicine kiosks, equipped with non-invasive biosensors, lead to significant health improvements in diabetes and hypertension patients, making it a viable alternative to (but not a replacement of) traditional hospital visits.

## 2. Literature Review

Telemedicine has been an area of significant research and development, with numerous studies exploring its various aspects and applications. For instance, Cilliers (2022) provided an in-depth analysis of the critical role of telemedicine in Africa, urging political leaders to take proactive measures to enhance its implementation [16]. Similarly, Indria et al. (2020) examined the perceptions of Indonesian physicians regarding existing telemedicine systems, finding that 78% were satisfied with text-based services despite challenges with internet connectivity [17]. To address these connectivity issues in remote areas, our study employed LoRa technology, which is known for its reliable long-range communication capabilities. This technology facilitates telemedicine in distant hospitals or health kiosks without internet access, thereby enhancing the accessibility and effectiveness of healthcare services in underserved regions.

In China, the implementation of telemedicine services in 2014 significantly improved the effectiveness of medical consultations and reduced costs [18]. However, these services did not incorporate relevant non-invasive sensors. Meanwhile, several studies have focused on wearable devices in telemedicine [19,20], but the high cost of devices like smartwatches makes them impractical for many Thai civilians [21]. Therefore, the introduction of telemedicine health kiosks offers a cost-effective alternative, eliminating the need for individuals to purchase expensive wearable devices. There are several approaches for Internet of Things (IoT) and machine learning models in biomedical applications [22,23,24,25,26,27]. However, none of those reported on the integration of noninvasive biosensors like noninvasive glucose sensor, time-series predictors, and long-range communications for NCD telecare using kiosks as well as medical proof of its impact on NCD conditions after the telemedicine system usage. A comparative analysis of various telemedicine implementations, highlighting their key features and relative advantages, is presented in Table 1.

Table 1 provides a comparative analysis of various telemedicine platforms, highlighting their key features and relative advantages. Teleconsultation and text-based telemedicine are low-cost solutions, primarily focusing on condition control without integrating non-invasive sensors or advanced AI prediction capabilities [17,18]. Wearable telemedicine for inflammatory bowel disease (IBD) and smartwatches for atrial fibrillation detection incorporate preventive care measures and non-invasive sensors but come with higher costs [19,21]. These platforms also lack long-range communication and advanced AI features. In contrast, the “Temed Suksala” system distinguishes itself by employing AI predictions using LSTM models, which will statistically analyze its significant contributions to NCD parameter improvements and long-range communications (LoRa) for distant kiosks. The real-time LSTM medical parameter predictions allow individuals to take care of themselves before the NCD parameters become worse. It also includes non-invasive biosensors for blood glucose, kidney injury biomarkers, and blood pressure monitoring. Furthermore, the system utilizes a priori knowledge discovery techniques to report to a civil governance officer, in order to address the root cause of geologically and occupationally correlated diseases. This comprehensive integration of technologies ensures condition control and preventive care at a very low cost, making it a promising solution for rural healthcare settings. The innovative approach of “Temed Suksala” addresses the limitations of existing telemedicine solutions, offering a robust, cost-effective alternative for improving healthcare accessibility in remote areas.

## 3. Methods

**Figure 2:**
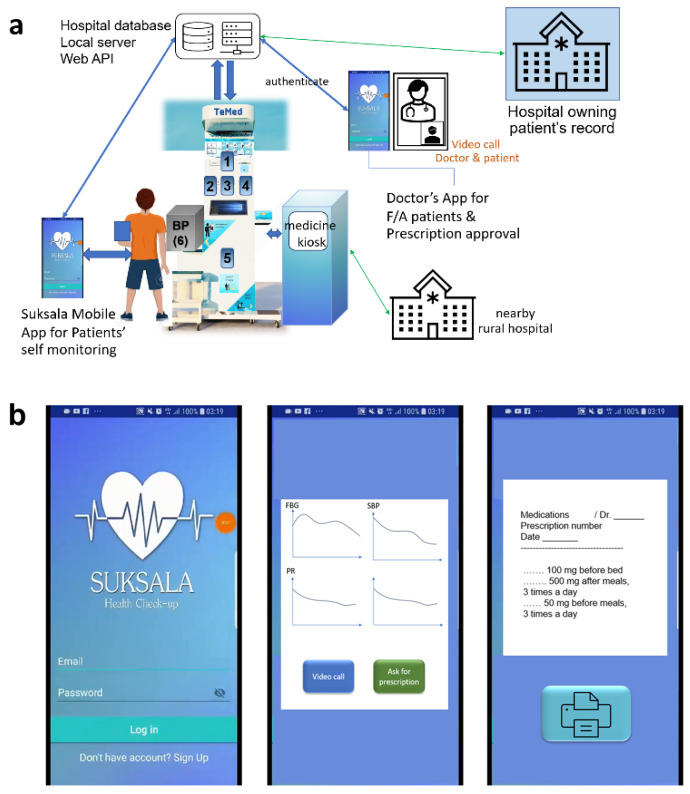
Illustration of (**a**) Suksala Temed embedded with a pharmacy kiosk. The kiosk consists of 1. non-invasive glucose sensors, 2. pulse oximeter, 3. temperature sensor, 4. smart card reader, 5. acute kidney injury (AKI) biomarker screener, and 6. blood pressure meter. A touch screen monitor with embedded camera was added for video calls and tele-prescription carried out by personal physician. (**b**) Mobile application screens, including login screen, record plots and predictions, and printable prescriptions from the doctor.

The addition of a medicine kiosk to the Temed (telemedicine) health kiosk to provide pills as tele-prescribed by a doctor is illustrated in Figure 2. Patients can follow their own records via either the Suksala mobile app or via the kiosk station, have video calls with their doctors, and receive approved prescriptions via telemedicine. Finally, they could grab the pills from the pharmacy kiosk attached to the health kiosk. For real-time parameter prediction, we used a long short-term memory (LSTM) model to predict and warn before severity. Cohort studies were randomly assigned to two groups: (i) telemedicine-using group and (ii) control group (without telemedicine), equally distributed with respect to age, diabetes symptoms, and hypertension symptoms. For the biosensor readings that contain missing data, we used interpolation replacement. The data preprocessing and AI prediction algorithm will be described in the following sections.

### 3.1. Long-Short Term Memories for Condition Prediction

Time-series predictors analyze historical and real-time data to forecast health trends and detect anomalies. This combination improves proactive disease management and prevention before the conditions become worse or critical. The long short-term memory (LSTM) algorithm is particularly suited for time-series predictions in telemedicine kiosks due to its ability to capture long-term dependencies in sequential data [20]. LSTMs address the vanishing gradient problem seen in traditional RNNs, facilitating effective learning from extended patient history. They adeptly model complex, non-linear relationships in physiological data, ensuring precise forecasts. Additionally, LSTMs handle noisy, irregularly sampled data common in real-world medical settings, making them robust and reliable for health trend predictions. This capability enhances early diagnosis and proactive disease management in telemedicine applications. LSTM contains key components: cell state, input gate, forget gate, and output gate. This enables LSTM to learn and forget information over time, making it effective at capturing time-series patterns. The cell state acts as memory and is updated through input, forget, and output gate operations. The input gate determines which information to store, the forget gate determines what to discard from the previous cell state, and the output gate controls the flow of the information to the next hidden state. LSTMs take past observations as inputs and forecast the next data point by capturing temporal patterns in the data. The LSTM time-series predictors for blood glucose and blood pressure were carried out under the following steps.

#### 3.1.1. Data Collection and Preprocessing

In this study, data were collected from 1844 volunteers using a telemedicine health kiosk. Blood glucose levels were measured non-invasively using a blood glucose sensor embedded in the kiosk, while systolic blood pressure readings were obtained using a standard pressure meter integrated within the same kiosk. For each individual, the first 10 measurements were collected to train the time-series predictor (LSTM) model.

##### A. Normalization

Normalization is an essential preprocessing step to ensure that the input features to the LSTM network are scaled appropriately. This helps in accelerating the convergence of the model and improving its performance. The normalization was performed using Min–Max scaling to cover the overshooting range. The Min–Max scaling equation is given by

$$X^{\prime }=\frac{X-X_{min}}{X_{max}-X_{min}},$$

where *X*^′^ is the normalized value, *X* is the original value, $X_{max}$ is the maximum value, and $X_{min}$ is the minimum value. Normalization could make LSTM training converge faster because the gradients during backpropagation are more stable and less likely to cause issues such as exploding or vanishing gradients, which are common problems in training deep neural networks, including LSTMs. In addition, normalization ensures that the features are on a similar scale, which is particularly important for LSTM networks, as they are sensitive to the scale of input data.

#### 3.1.2. LSTM Model

**Figure 3:**
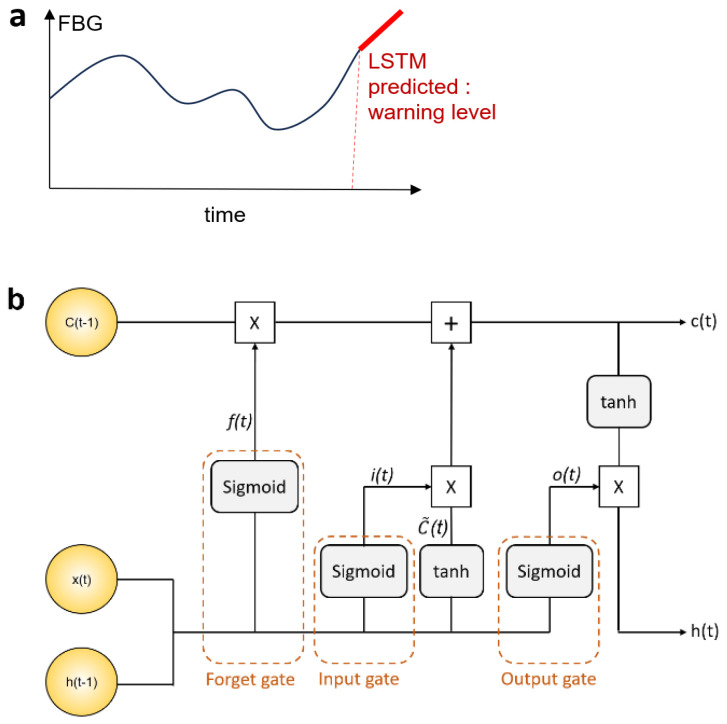
(**a**) Prediction of blood glucose level and warning via LSTMs. (**b**) Diagram of LSTM layer or LSTM algorithm.

In this study, LSTM was applied to predict NCD conditions, such as predicting and warning before real-time blood glucose levels become too high (Figure 3). The prediction system could tell the patient to handle their glucose level (and other parameters) carefully, thus yielding improvements in their overall condition.

The LSTM prediction algorithm is summarized in Figure 3 and the equations are as follows:

(1) $$f\left(t\right)=\sigma (W_{f}\cdot \left[h\left(t-1\right),x\left(t\right)\right]+b_{f}$$

(2) $$i\left(t\right)=\sigma (W_{i}\cdot \left[h\left(t-1\right),x\left(t\right)\right]+b_{i})$$

(3) $$o\left(t\right)=\sigma (W_{O}\left[h\left(t-1\right),x\left(t\right)\right]+b_{O})$$

where *x*(*t*) denotes the latest input at time step *t*, and *i*(*t*), *f*(*t*), and *o*(*t*) are the activations of the input, forget, and output gates, respectively, that control information flow. *σ* is the sigmoid function. *h*(*t*−1) and *c*(*t*−1) are the hidden state and cell state of the LSTM at the previous time step *t* − 1. *W_i_*, *W_f_*, and *W_o_* are the weight matrices for the input, forget, cell, and output gates, respectively. *b_i_*, *b_f_*, and *b_o_* are the bias vectors for the input, forget, cell, and output gates, respectively. The cell and hidden states were computed using the following equations:

(4) $$\tilde{C}\left(t\right)=tanh(W_{C}\cdot \left[h\left(t-1\right),x\left(t\right)\right]+b_{C}),$$

(5) $$C\left(t\right)=f\left(t\right)*C\left(t-1\right)+\left(1-f\left(t\right)\right)*\tilde{C}(t),$$

(6) $$h\left(t\right)=o\left(t\right)*\tanh\left(C\left(t\right)\right),$$

where $\tilde{C}$(*t*) denotes the candidate state added to *i*(*t*)*. W_C_* and *b_C_* are the weight matrix and the bias vector of the cell state, respectively. *h*(*t*) and *C*(*t*) represent the hidden and cell states, respectively, for the next cycle.

#### 3.1.3. Model Training and Validation

An LSTM network was designed to predict blood glucose and systolic blood pressure from temporal data. The architecture included input, LSTM, and dense layers. Backpropagation through time (BPTT) was used for gradient computation. Adam (adaptive learning rate optimization algorithm) optimizer was used for training or weight adjustment. Data from 1844 volunteers was split according to chronological train–validation split: (i) a training set consisting of the earlier part of the time series data (using the first 25 measurements), and (ii) a validation set consisting of the latter part of the time series data (we used the next 10 measurements), following the training set. After training, the model was applied and adjusted throughout the 8-month time series. The model used early stopping to avoid overfit. Before training can start, we need to define the metric to measure the difference between actual and predicted values, i.e., the loss function. The loss function used in the model was Mean Squared Error (MSE), which is typically used for regression metrics to measure the difference between the predicted and actual values. MSE took advantage of having the gradient due to its differentiable property over other metrics, like absolute error. $MSE=\frac{1}{n}\sum_{i=1}^{n}{\left(y_{i}-{\hat{y}}_{i}\right)}^{2}$ where $y_{i}$ is the actual value, ${\hat{y}}_{i}$ is the predicted value, and *n* is the number of samples. The training and validation processes are described as follows.

The training process for LSTMs using Adam and BPTT begins with data preparation, involving sequence padding and normalization. The LSTM network is initialized with random weights. In each training epoch, mini-batches of sequences are fed through the network. BPTT computes gradients by propagating errors backward through time steps. These gradients are then passed to the Adam optimizer, which calculates adaptive learning rates for each parameter and updates the weights accordingly. This process repeats for multiple epochs, with the model’s performance monitored on a separate validation set to prevent overfitting.

Validation is performed periodically during training, usually after each epoch. A separate set of sequences, not seen during training, is used to evaluate the model’s generalization ability. The LSTM processes these validation sequences in a forward pass only, without updating weights. Performance metrics such as mean squared error or accuracy are computed on the validation set. If validation performance plateaus or degrades while training performance continues to improve, early stopping may be implemented to prevent overfitting. The learning rate may also be adjusted based on validation performance, either manually or through techniques like learning rate scheduling.

- Gradient Computation: BPTT in LSTMs computes gradients by first performing a forward pass through all time steps, storing activations and cell states. The backward pass then starts from the last time step, calculating error gradients for outputs, LSTM gates, cell states, and weight matrices at each step. Gradients are accumulated across all time steps for each parameter, often with gradient clipping to prevent explosions. The LSTM’s gating mechanism aids in propagating gradients through long sequences, though very long dependencies can still be challenging. While computationally intensive for long sequences, optimizations like truncated BPTT can be applied. This process enables LSTMs to learn from temporal data by adjusting weights based on errors propagated through time, allowing the network to capture both short-term and long-term dependencies in the input sequences.
- Parameter Update: The Adam optimizer uses these gradients to update the parameters (weights and biases) of the LSTM model. It adjusts the learning rates for each parameter based on the estimates of first and second moments of the gradients, which helps in faster convergence and better handling of different types of data and architectures.
- Efficient Training: Together, BPTT and Adam allow the LSTM model to efficiently learn from sequential data. BPTT provides the mechanism to propagate gradients back through time to capture long-term dependencies, while Adam optimizes the learning process by adjusting the model parameters effectively.

An LSTM network was designed to predict blood glucose and systolic blood pressure using temporal data from 1844 volunteers, split into training and validation sets based on chronological order. The network, comprising input, LSTM, and dense layers, was trained with BPTT and the Adam optimizer, utilizing MSE as the loss function. Early stopping and gradient clipping were employed to prevent overfitting and manage gradient explosions. Validation was performed periodically, and the model was adjusted throughout an 8-month time series to ensure efficient learning and capture of long-term dependencies.

### 3.2. Design and Implementation of Suksala Telemedicine

#### 3.2.1. Network of Sensors

Multiple Temed health kiosks equipped with non-invasive sensors can form a wireless medical sensor network. Data records were stored locally in a rural hospital hub. LoRa was used for remote areas where the kiosk was located 1.5–4 km away from the nearest communication hub, providing telemedical services to individuals in rural regions. To overcome serial connection instability issues within the Temed Health Kiosk, local sensor information is transmitted using Message Queuing Telemetry Transport (MQTT) protocol, which is a lightweight, publish-subscribe-based communication method with a local broker utilizing a Raspberry Pi 4B. For distant communications, the system selects 5G, IEEE 802.11, or LoRa, depending on the distance and availability of the signals.

#### 3.2.2. Sensor Details

**Figure 4:**
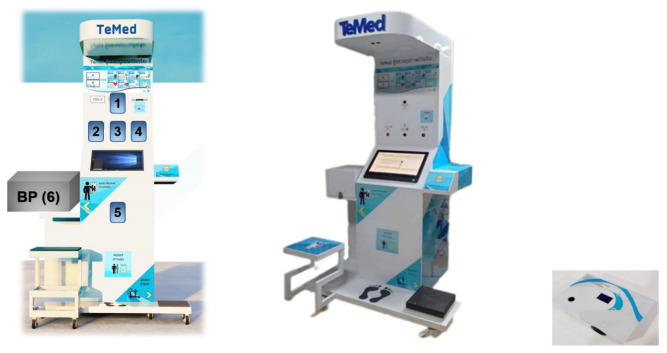
(**left**) Illustration of non-invasive sensors embedded in the “Temed Suksala” telemedicine kiosk. There are several components: (1) non-invasive glucose sensor (developed), (2) pulse oximeter (standard product available), (3) temperature sensor, (4) gas sensors, (5) AKI screener from urine sample [14,15], (6) standard blood pressure sensor and other traditional sensors such as ultrasonic height sensor and strain-gauge weight sensor. (**middle**) A real image of the Suksala Temed kiosk. (**right**) An embedded non-invasive glucose sensor [13].

For non-invasive glucose sensing, we implemented an AI-enhanced multiband infrared sensor that achieves accurate blood sugar level determination. The accuracy and performance of this method are clinically acceptable, as reported by Srichan et al. (2022) [13]. For non-invasive AKI biomarker sensing, we utilized 3D microporous graphene, which is known for its high sensitivity, along with enzyme-coated surfaces to provide specificity. Amperometric detection of human urine to determine neutrophil gelatinase-associated lipocalin (NGAL), the most rapid kidney damage indicator, was performed as described in [14,15]. Other sensors, such as smart card readers, height sensors, load cell weight sensors, and pulse oximeters, were procured, calibrated, and integrated into the kiosk. In addition to non-invasive sensors, the system also provides video calls between doctors to investigate the symptoms. Subsequently, the doctor prescribed compulsory medicine, which the patient could claim from the nearby medical kiosk in sync with the nearby rural hospital. The kiosk with integrated sensors are shown in Figure 4.

### 3.3. Internetworking of Suksala Telemedicine Kiosks

We deployed the Suksala telemedicine health kiosks at five stations in northeast Thailand. Credential information is encrypted and transmitted through multiple channels. The communication protocols used were IEEE 802.11n (2.4 GHz Wi-Fi), 5G, and LoRa, selected based on internet connection availability and the distance from the kiosk to the nearest internet connectable areas. The WiFi we used was the built-in WiFi 2.4 GHz module. 5G module was manufactured by Quectel, Shanghai, China. For the areas where Wi-Fi or 5G were available, we used either one of them for the kiosk communication with the cloud API. To communicate to and from a telemedicine kiosk situated in distant (1.5–4 km), rural areas with no signal, we employed LoRa (923 MHz), which is a permitted frequency band in the country. One end was the kiosk in a distant area with no signal. The other end was the nearest rural hospital where Wi-Fi connection is also available. The hardware implemented was LoRa transceiver modules connected with ESP32 microcontrollers (developed by Espressif Systems and manufactured at TSMC, Hsinchu, Taiwan). One end, which receives signals from the kiosk, has a gateway implemented using Raspberri Pi 4B (manufactured by the Raspberry Pi Foundation in association with Broadcom, Cambridge, UK) as hardware. The ThingGateway was deployed on a Raspberry Pi, which involved installing the required software, configuring the gateway and MQTT connector, and setting up the system to start the gateway automatically on boot. Once configured, the Raspberry Pi will communicate with the ThingsBoard server, enabling data collection from a connected LoRa node, the distant kiosk node. 

### 3.4. Software Architecture

#### Patient Access to the Telemedicine Kiosk

To access the Temed kiosk service, patients insert their national identification card into a smart card reader for identity verification, supplemented by facial recognition using wavelet features and deep neural networks. Patient records are stored locally in the kiosk and hospital databases, ensuring compliance with national privacy regulations by avoiding cloud storage. Patients use their citizen ID and a password to access their health history via mobile applications, allowing them to review their health conditions and recommendations. Physicians access the system through username/password authentication with role-based access control, which may include additional security measures like biometrics and one-time passwords based on hospital regulations.

### 3.5. Health Record Structure of Temed Suksala Kiosks

The health record from the kiosk comprises (i) patient ID; (ii) personal information (name, surname, date of birth, and occupation); (iii) readings from non-invasive sensors (glucose, blood pressure, pulse rate, SpO_2_, NGAL, and BMI); (iv) diagnosis results by doctors; (v) latest prescriptions; and (vi) date and time stamp. We followed the architecture of Fast Healthcare Interoperability Resources (FHIR) to handle the patients’ records between the kiosk, mobile application, and local hospital server. The records were customized to the measurement variables provided by the non-invasive sensors on the Temed health kiosk.

### 3.6. Improvement of Fasting Blood Glucose (FBG) and Systolic Blood Pressure (SBP) via Statistical Proof

There are four hypotheses for each parameter. For FBG, there are two main groups: control group (without telemedicine) and the telemedicine kiosk-using group. For each group, we divided into two temporal groups, i.e., before and after 8-month usage (or non-usage) of the telemedicine kiosk. These groups will be under statistical estimation for the kiosk impact on NCD conditions as follows.

#### 3.6.1. The Experimental (Telemedicine) Group

The paired *t*-test for the experimental (telemedicine-using) groups is defined as:

- H0 (Null hypothesis): The mean fasting blood glucose (FBG) levels are not significantly different before (G1) and after (G2) 8 months of telemedicine usage.

$$H0:\mu_{G2}-\mu_{G1}=0$$

- H1 (Alternate hypothesis): The mean fasting blood glucose (FBG) levels are significantly different before (G1) and after (G2) 8 months of telemedicine usage.

$$H1:\;\mu_{G2}-\mu_{G1}\neq 0$$

Note that according to the collected data, $H1:\;\mu_{G2}-\mu_{G1}\neq 0$ implies that $\mu_{G2}-\mu_{G1}<0$, or a significant improvement of fasting blood glucose in the telemedicine-using group, where $\mu_{G1}$ is the mean FBG at the first visit (before telemedicine), and $\mu_{G2}$ is the mean FBG after 8 months (after telemedicine).

#### 3.6.2. The Control Group (without Telemedicine)

For the control group, the paired *t*-test hypotheses are as follows:

- HC0 (Null hypothesis): The mean fasting blood glucose (FBG) levels are not significantly different before (C1) and after (C2) 8 months without telemedicine usage.

$$HC0:\;\mu_{C2}-\mu_{C1}=0$$

- HC1 (Alternate hypothesis): The mean fasting blood glucose (FBG) levels are significantly different before (C1) and after (C2) 8 months without telemedicine usage.

$$HC1:\mu_{C2}-\mu_{C1}\neq 0$$

where $\mu_{C1}$ is the mean FBG at the first visit (control group, before 8 months), and $\mu_{C2}$ is the mean FBG after 8 months (control group, after 8 months).

#### 3.6.3. Statistical Proof and Analysis

To prove the improvement in FBG via statistical proof, follow these steps. A. Collect data: Measure FBG levels for both groups (experimental and control) at the start of the study and after 8 months. B. Calculate Mean Differences: For the experimental group, $DE=\mu_{G2}-\mu_{G1}$. For the control group, $DC=\mu_{C2}-\mu_{C1}$. C. Perform Paired *t*-tests: Conduct a paired *t*-test to compare the FBG levels before and after 8 months for (i) experimental (telemedicine) group and (ii) control group, before and after 8 months without using telemedicine kiosk.

D. Interpretation method

(1) If the *p*-value for the experimental group is less than the significance level (0.05), reject the null hypothesis H0 and conclude that there is a significant improvement in FBG levels with telemedicine usage.
(2) If the *p*-value for the control group is less than the significance level (0.05), reject the null hypothesis HC0 and conclude that there is a significant difference in FBG levels without telemedicine usage.

Similar arguments will be used for the systolic blood pressure. We will arrive at a conclusion whether using the telemedicine kiosk system helps in significant improvements of NCD conditions in terms of fasting blood glucose and systolic blood pressure.

### 3.7. Association Rules

The symptoms and significant diseases in each sub-district of Khon Kaen are shown in the tables and figures. Possible correlation events for each major symptom in every area were discovered by association rule finding using the Apriori algorithm on the big data of spatial land environments, underground materials, the majority occupation of people, and related symptoms. The association rules resulting from big data mining are presented in Table 2.

The Apriori algorithm was used to determine this association. This process can be divided into the following steps:

(1) Data Preparation: People visited the Temed health kiosks several times a week. They took non-invasive measurements of blood glucose, pulse rate, blood pressure, and other parameters. A person’s citizen ID and latest health data were recorded in the local MongoDB database. NoSQL was used because some information, such as complaints in the form of text, voices, or videos, was not structured. The calculations are summarized below.
(2) Support (*S*) computation: The support of an itemset *X* is given by

*S*(*X*) = (#transactions containing *X*)/(#total transactions), (7)

(3) Candidate Generation: The Apriori algorithm generates candidate itemsets of size *k* + 1 based on frequent itemsets of size *k*. For example, it generates candidate 2-itemset (*C*2) based on frequent 1-itemset (*L*1), candidate 3-itemset (*C*3) based on frequent 2-itemset (*L*2), etc.
(4) Apriori Property: The Apriori property is used to efficiently reduce the search space of candidate itemsets. It states that if an itemset is infrequent, then all its supersets will also be infrequent. This property allows the algorithm to avoid unnecessary candidate generation and pruning, thereby increasing its efficiency.
(5) Frequent Itemset (*L*): Frequent itemsets were defined as those with a support greater than or equal to the minimum support threshold (min_sup_). The itemset was considered meaningful and potentially significant for generating association rules.
(6) Confidence calculation (*C*): Confidence measures the strength of association rule X→Y. It is calculated as the support of the combined itemset (*X* ∪ *Y*) divided by the support of the precedent itemset (*X*). Mathematically, the confidence of rule X→Y is given by:
  (8) $$C(X\to Y)=S\left(X\;U\;Y\right)/S(X)$$

Based on these steps, the Apriori algorithm efficiently discovers frequent itemsets in large transactional datasets and generates association rules that meet the specified minimum support and confidence thresholds. We selected high-confidence association rules and report them in Table 2.

## 4. Results

### 4.1. Statistical Assessment Whether Telemedicine Kiosk Improves NCD Conditions

**Figure 5:**
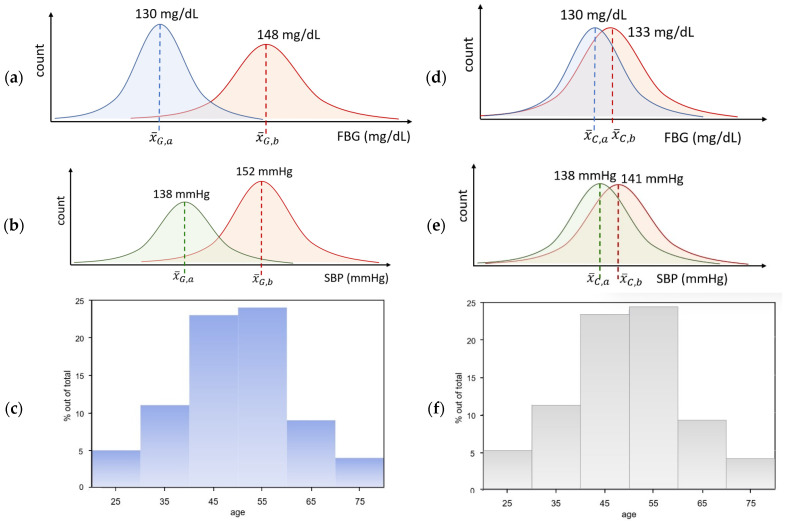
Telemedicine-using group and control group compared. (**a**,**b**) Improvement results of each NCD condition after long-term 8-month visiting of Temed in the same area. A total of 1844 volunteers were recorded for the telemedicine-using group, and their conditions were improved; they are illustrated by comparing averaged fasting blood glucose (FBG) and resting body systolic blood pressure (SBP). (**c**) Age distribution ratio of all volunteers. (**d**,**e**) Nonsignificant improvement results in the control group (without telemedicine kiosk usage). (**f**) Age distribution of the control group (N = 1210). G represents telemedicine group and C denotes the control group, where a and b denote before and after 8 months.

Figure 5a,b show the improvement of NCD conditions after telemedicine kiosks were implemented for 8 months. In a 7-day window, average fasting blood glucose and average systolic blood pressure were recorded and compared. Age distributions are illustrated in Figure 5c. Improvement of SBP and FBG can be proved via *t*-test statistics.

In the telemedicine-using set, a paired *t*-test was conducted to assess the significance of the difference in the mean fasting blood glucose levels between group 1 (G1) at month 0 (mean = 148) and group 2 (G2) (mean = 130). The null hypothesis, stating that there was no significant difference in mean fasting blood glucose levels between the two groups, was tested against the alternative hypothesis, which posits a significant difference. The analysis revealed a statistically significant difference in the mean fasting blood glucose levels, t(DF) = 2.5, *p* = 0.04 (where DF is the degree of freedom). The significance level was set at α = 0.05. Because *p* = 0.04 < α, we reject the null hypothesis. Therefore, the average FBG levels in the two groups were significantly different. Because the average FBG after 8 months passed was better (lower) than the average FBG in the first month, we concluded that there was a significant improvement in the average fasting blood glucose levels after the 8-month implementation of telemedicine. Similar arguments can be made regarding SBP improvement.

For a paired *t*-test over the control group (no telemedicine kiosk), with a significance level of 0.05, we fail to reject the null hypothesis (*p* = 0.1 > 0.05), indicating that there is no statistically significant difference between the mean values of group C1 and C2. Therefore, we conclude that the two groups are statistically indifferent in terms of 8-month ordinary care without telemedicine in the rural population.

Eight months after the telemedicine kiosk operation, the symptoms of patients with NCD improved according to several indicators. The mean fasting blood glucose (FBG) levels improved by 18 mg/dL in volunteers (Figure 5a), SD = 4.5 mg/dL. The average systolic blood pressure was better by 14 mmHg (Figure 5b) (SD = 3.6 mmHg. These statistics were calculated for the entire population that underwent measurements (Figure 5c). For the elderly group (>50 years old), the difference was 40% less than the previous statistics; that is, blood glucose improved by 11 mg/dL and systolic blood pressure for HT patients was reduced by 8.5 mmHg. However, acute kidney injury (AKI) detection through neutrophil gelatinase-associated lipocalin (NGAL) measurement was not different in the group of study participants. A urine NGAL level greater than 100 ng/L indicates a potential case of AKI, and further assessment by a nephrologist is necessary for confirmation. In this study, urine NGAL levels were determined using electrochemical methods [13,14]. Patients with hypertension were said to have improved when they could control their resting body systolic blood pressure to improve across the ranges (≥140 stage 2 high, 130–139 stage 1, 120–129 elevated normal, and 110–120 normal). The pulse rate was considered to improve if it moved from >100 bpm or <60 bpm to 60–100 bpm, without an arrhythmic pattern. These could be measured when people visited the telemedicine kiosk; however, only blood glucose and blood pressure were regularly recorded by the volunteers.

### 4.2. Knowledge Discovery Result

**Figure 6:**
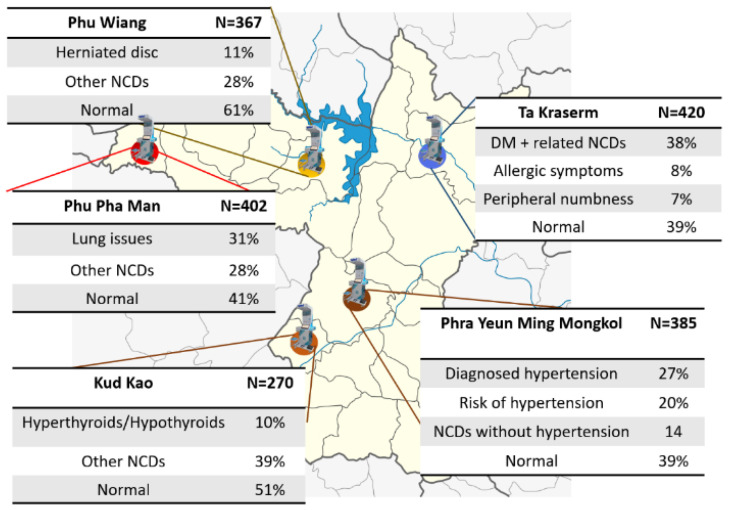
Map of subdistricts in Khon Kaen where Temed health kiosks were distributed, and the diseases/symptoms are summarized in each area. Map figure adapted from Heinrich Damm under CC-BY 3.0 license.

The mined association rules indicate the relationships among occupation, environment, and ailment conditions. For example, hyperthyroidism and hypothyroidism were found in the area beneath rock mountains with mineral-rich water resources. In villages near rivers, their farms yield enormous amounts of fruits and vegetables despite the high usage of herbicides and pesticides. The health of these patients is affected when they develop allergic symptoms and peripheral numbness. In the meantime, farmers who live near rivers and do not frequently use herbicides or pesticides do not develop allergies but herniate disks instead. For villagers living near stone mills or construction sites, lung conditions are not good, and SpO_2_ is between 94 and 97%, which is less than that in healthy people. Individuals in areas with high-salinity groundwater tended to develop hypertension at a confidence level of 47%. These facts were discovered after the gathered telemedicine data were analyzed, and the data-mining algorithm went through geographical information (Figure 6). The extraction of causes and illnesses addresses the root cause, which can be appropriately handled by the government.

The Apriori algorithm examines correlations between disease, geolocation, and occupation. For instance, identifying high-salinity areas correlated with hypertension could prompt policymakers to intervene by reducing salinity in soils and water. This proactive approach addresses root causes, preventing symptomatic treatment and illustrating digital governance policies for public health in the region.

### 4.3. Field Results

**Figure 7:**
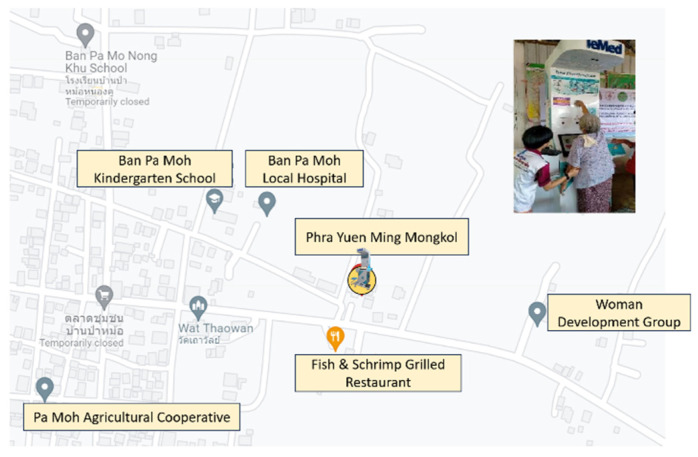
Illustration of Phra Yuen Ming Mongkol subdistrict, where the telemedicine kiosks were delivered. In this area, we found that 47% of villagers developed hypertension and the groundwater’s salinity is higher than normal. Location = (16.3253034, 102.6035327).

**Figure 8:**
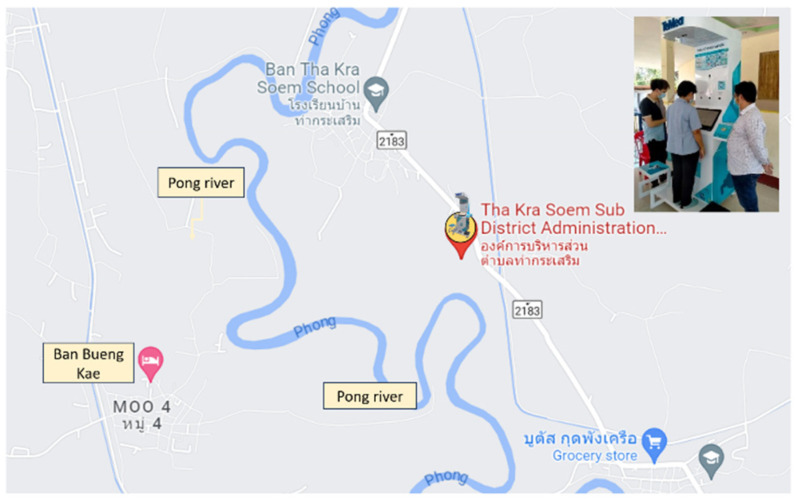
Tambol Ta Kraserm, where the significant discovery was either allergic symptoms or peripheral numbness. Note that there was high usage of pesticides and herbicides in agriculture in this area. Location = (16.6071006, 102.8487543).

Large data collections and mining of the patient’s history and health records, along with the available information, are summarized in Table 3. Before running the Apriori algorithm, the two databases—areal information and patient records—were merged using the citizen ID. The results showed that in the first area, Phra Yuen District, 47% of the villagers had hypertension or were developing hypertension, and the groundwater showed high salinity. In the Tha Kra Soem sub-district, 15% of the population developed either allergies or peripheral numbness. Herbicides, pesticides, and chemical agents have been extensively used in this area. In the Phu Pha Manh district, 31% of people were found to develop lung issues, and stone mills were located nearby. In the Kud Kao sub-district of Mancha Kiri, villagers develop hyperthyroidism and hypothyroidism. This may have been correlated with the high mineral content of the nearby swamps. Figure 7 and Figure 8 show the distribution of Suksala Temed kiosks in different areas.

## 5. Discussion

Overall, a smart city with an integration of telemedicine services, non-invasive sensors, and AI prognosis predictors/warning systems represents a promising direction for healthcare innovation and could significantly improve patient care and reduce healthcare costs. There are at least five advantages of this study in comparison with other trending reports on telemedicine implementation. 

First, wearable smart devices are designed only for personal use. Thus, it is too expensive for most people in low- to middle-income countries. Second, the telemedicine kiosk could reduce transportation costs of visiting the hospital frequently and medications, owing to the improvement in health conditions via AI prediction and rapid general guidelines. Third, it reduces congestion in the hospital, thus reducing the risk of exposing patients to coronaviruses and any life-threatening communicable diseases that may exist in the future. Fourth, preventive care should focus on effective treatments. People can become alarmed by AI, such as when blood glucose is going to be high in the next hour, and must take proper action depending on the conditions. If a patient already has NCDs, such as diabetes, they could also receive an AI warning based on their history of non-invasive glucose measures and receive proper care to achieve better conditions, such as a controllable glucose level and better blood pressure, due to less diabetes-induced endothelial damage. Finally, it is cheaper for patients to arrive and use the health kiosk service than to force civilians to buy smart wearable devices. 

Based on a study of 1844 volunteers, approximately 30% improved after eight months of service at the telemedicine kiosk. A paired *t*-test was conducted to prove the hypothesis that average SBP and FBG levels improved after the implementation of telemedicine. The association rules between symptoms/diseases, occupation, and geospatial location have also been studied and reported. This is a promising result and could pave the way for further integration, improvement, and distribution of the system to other areas in Thailand and other countries of interest.

The Apriori algorithm analyzes correlations between disease, geolocation, and occupation. For instance, identifying hypertension in high-salinity areas could inform targeted interventions to reduce salinity, thereby addressing root causes rather than symptoms, and serving as a model for digital governance policies in public health.

However, the proposed framework has some limitations. Even though the telemedicine kiosk provides more healthcare accessibility and preventive care via real-time prediction before critical parameters arise, it cannot handle severe case where the patient cannot interact with the kiosk and when the available sensors are not sufficient for diagnosis and monitoring, for example, when an X-ray is needed for fractured bones, etc.

## 6. Conclusions

The design and implementation of a smart city with a telemedicine system embedded with non-invasive sensors, AI predictions, and big data knowledge discovery were carried out and reported in this work. The telemedicine system led to a statistically significant improvement in fasting blood glucose from 148 to 130 mg/dL (mean reduction of 18 mg/dL, SD = 4.5 mg/dL) and systolic blood pressure from 152 to 138 mmHg (mean decrease of 14 mmHg, SD = 3.6 mmHg). Meanwhile, the control group (without telemedicine) showed nonsignificant difference (a little worse) in the NCD care of fasting blood glucose and blood pressure after 8 months. These results demonstrate a clinically meaningful improvement with the implementation of the telemedicine system. With a telemedicine system, hospital congestion and the risk of airborne infections can be reduced to arbitrary levels. Although smart wearable devices have been proposed for partial telemedicine tasks, they are expensive and infeasible for large populations. If telemedicine kiosks could be extended to serve more people than in the current study, enormous national costs in time and transportation to and from hospitals could be reduced. Improvements in health can also reduce the cost of medication. Synchronization with large hospitals could be further improved to expand the use of health kiosks for the tele-referral of patients before arrival at the hospital. In our future work, more non-invasive sensors related to NCDs could be added to increase functionality and cover more diseases. The results are promising and could assist smart health policymakers in making proper decisions towards a fully functioning smart city. Extending this work would, in turn, promise a brighter future for smart cities with smart healthcare integration.

## Figures and Tables

**Table 1:** Comparison of different telemedicine systems.

Platform	Long-Range Communications	AI Prediction	Knowledge Discovery	Preventive Care	Condition Control	Cost	Include Non-Invasive Sensors	Reference
Teleconsultation	×	×	×	×	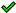	low	×	Cui et al. (2021) [18]
Text-based telemedicine	×	×	×	×	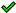	low	×	Indria et al. (2020) [17]
Wearable telemedicine in IBD	×	×	×	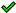	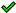	high	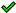	Rowan & Hirten (2022) [19]
Smart watch for Atrial fibiliation detection	×	×	×	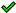	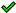	high	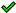	Perez et al. (2019) [21]
“Temed Suksala” with non-invasive sensors	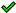 (LoRa)	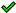 (LSTMs)	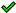 (Apriori discovery)	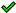	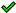	Very low	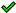	This work

**Table 2:** Association rules from Apriori algorithm.

Association Rules (Most Relevant Rules Shown)
1. hyperthyroid_or_hypertyroid=1 61 ==> near_river=1 near_corrosive_rock_mountain=1 60 <conf:(0.98)> *
2. near_river=1 abundant_agricultural_resource=1 herbicides_or_pesticides_usage=1 is_patient=1 490 ==> allergy=1 OR peripheral_numbness=1 OR other_NCDs=1 486 <conf:(0.92)>
3. near_river=1 abundant_agricultural_resource=1 herbicides_or_pesticides_usage=0 is_patient=1 541 ==> herniated_disc=1 OR other_NCDs=1 537 <conf:(0.92)>
4. near_stone_mill=1 OR near_construction_site=1 is_patient=1 450 ==> lung_health_issues OR other_NCD 140 <conf:(0.31)>
5. high_salinity_in_soil=1 is_patient=1 640 ==> HT=1 OR risk_of_HT 300 <conf:(0.47)>
6. high_salinity_in_soil=1 is_patient=1 640 ==> normal=1 250 <conf:(0.40)>

* Note that conf refers to the confidence level of rules 0–100%. Here, 1 and 0 refer to true or false, respectively.

**Table 3:** Significant symptoms found in different locations and their possible correlated events.

Area	Characteristics	Significant Diseases/Symptoms	Possible Correlation
(A) Phra Yuen	Lowland area	Hypertension	High water salinity
(B) Tha Kra Soem	Near Pong river	Skin allergy, numbness at hands and feet	High usage of herbicides/pesticides
(C) Phu Wiang	Near Pong river	Herniated disks	Farming profession
(D) Phu Pha Manh	Area near mountains and a stone mill	Lung and respiratory symptoms (low SpO_2_ compared to other areas), Allergy	Dust in the area, chemicals usages in agriculture
(E) Kudkao	near swamp	Hyperthyroid or hypothyroid	Near river

## Data Availability

Raw data cannot be made public for privacy and ethical reasons but will be available on reasonable request.
